# Poly[μ-aqua-μ_4_-terephthalato-strontium]

**DOI:** 10.1107/S1600536810054486

**Published:** 2011-01-29

**Authors:** Lei Yang, Dan Zhao, Guanghua Li

**Affiliations:** a The Department of Physics–Chemistry, Henan Polytechnic University, Jiao Zuo 454150, People’s Republic of China; b State Key Laboratory of Inorganic Synthesis and Preparative Chemistry, College of Chemistry, Jilin University, Changchun 130012, People’s Republic of China

## Abstract

In the title compound, [Sr(C_8_H_4_O_4_)(H_2_O)]_*n*_, the Sr^II^ atom exhibits coordination number eight, with six O atoms from four carboxylate groups (two bidentate and two monodentate) of  terephthalate ligands and two water O atoms. The SrO_8_ polyhedra are linked into inorganic chains by sharing three coplanar O atoms. These inorganic chains are extended along the *b* axis to form layers in the *ab* plane by O—C—O linking. Parallel layers are connected by terephthalic groups, forming a three-dimensional framework. O—H⋯O hydrogen-bonding inter­actions are observed.

## Related literature

For hybrid inorganic-organic framework materials, see: Férey *et al.* (2008 ▶); Zhang *et al.* (2009 ▶). 
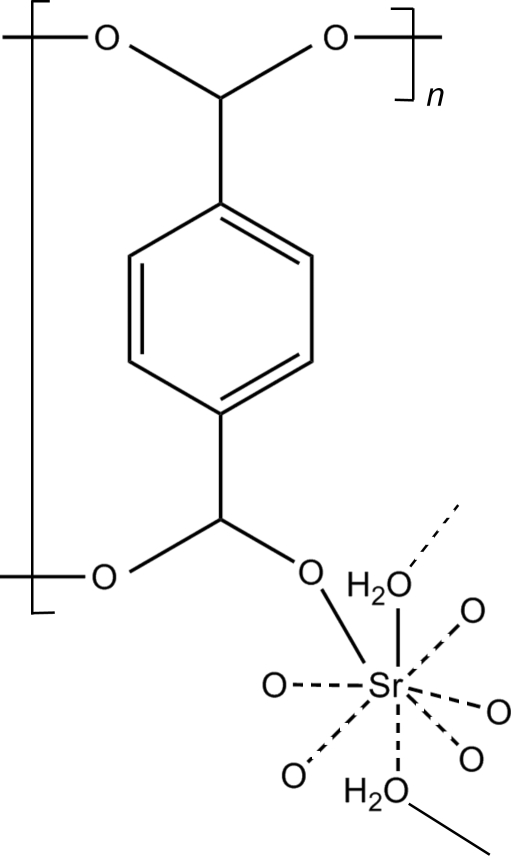


## Experimental

### 

#### Crystal data

[Sr(C_8_H_4_O_4_)(H_2_O)]
*M* *_r_* = 269.75
Orthorhombic, 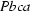

*a* = 11.8724 (3) Å
*b* = 7.1308 (1) Å
*c* = 20.0592 (4) Å
*V* = 1698.21 (6) Å^3^
*Z* = 8
Mo *K*α radiation
μ = 6.34 mm^−1^
*T* = 296 K
0.24 × 0.21 × 0.19 mm

#### Data collection

Bruker APEXII CCD diffractometer
Absorption correction: multi-scan (*SADABS*; Bruker, 2001 ▶) *T*_min_ = 0.238, *T*_max_ = 0.300
6767 measured reflections
1523 independent reflections
1205 reflections with *I* > 2σ(*I*)
*R*_int_ = 0.043

#### Refinement

*R*[*F* ^2^ > 2σ(*F* ^2^)] = 0.027
*wR*(*F* ^2^) = 0.062
*S* = 1.04
1523 reflections
133 parameters
3 restraints
H atoms treated by a mixture of independent and constrained refinement
Δρ_max_ = 0.36 e Å^−3^
Δρ_min_ = −0.50 e Å^−3^

### 

Data collection: *APEX2* (Bruker, 2001 ▶); cell refinement: *SAINT* (Bruker, 2001 ▶); data reduction: *SAINT*; program(s) used to solve structure: *SHELXS97* (Sheldrick, 2008) ▶; program(s) used to refine structure: *SHELXL97* (Sheldrick, 2008) ▶; molecular graphics: *SHELXTL* (Sheldrick, 2008) ▶; software used to prepare material for publication: *SHELXTL* ▶.

## Supplementary Material

Crystal structure: contains datablocks I, global. DOI: 10.1107/S1600536810054486/bx2340sup1.cif

Structure factors: contains datablocks I. DOI: 10.1107/S1600536810054486/bx2340Isup2.hkl

Additional supplementary materials:  crystallographic information; 3D view; checkCIF report

## Figures and Tables

**Table 1:** Hydrogen-bond geometry (Å, °)

*D*—H⋯*A*	*D*—H	H⋯*A*	*D*⋯*A*	*D*—H⋯*A*
O5—H1⋯O3^i^	0.84 (3)	2.03 (4)	2.711 (3)	137 (3)
O5—H2⋯O2^ii^	0.84 (3)	1.92 (3)	2.761 (3)	178 (3)

Symmetry codes: (i) 
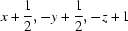
; (ii) 
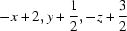
.

**Fig. 1.**
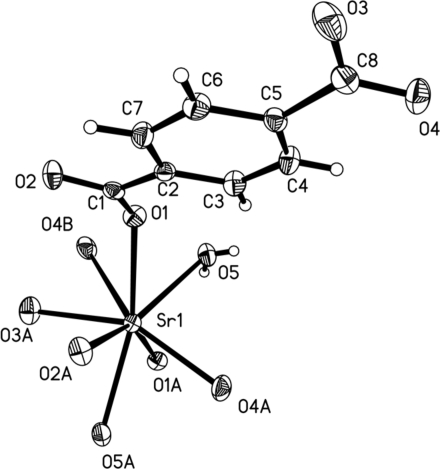
Asymmetric unit of compound with thermal ellipsoids.Displacement ellipsoids are drawn at the 50% probability level.

**Fig. 2.**
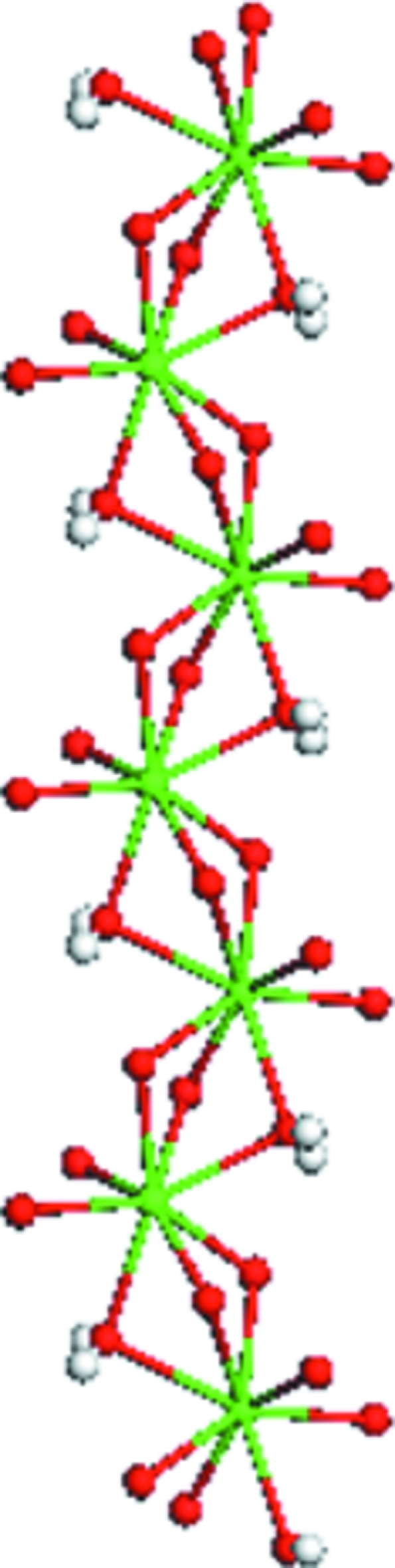
Sr polyhedra extended along the b axis to form one-dimensional chain by sharing three co-planar O atoms

**Fig. 3.**
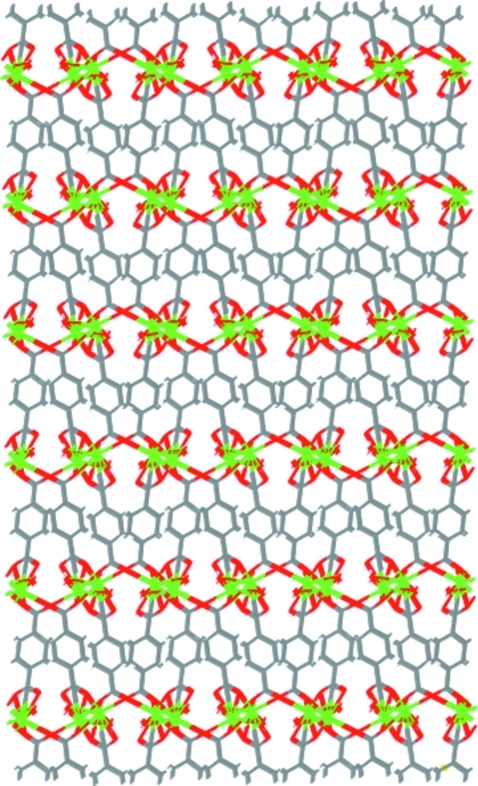
View of the structure along [0 0 1] direction, layers connected by terephthalic groups forming the three-dimensional framework

## supplementary crystallographic information

### Comment

Many researchers have focused their attention on the preparation and investigation of hybrid inorganic-organic framework materials because of their intriguing network structures, novel topologies, and potential applications, such as catalysis and optical materials. However the reports about hybrid inorganic-organic frameworks in lead coordination compounds are still less.n this paper, we described the synthesis and crystal structure of a novel hybrid inorganic-organic framework [Sr(C_8_H_6_O_5_)]_n_. Sr(II) atom in asymmetric unit are octahedrally coordinated(Fig1) which is coordinated by six oxygen atoms from terephthalate and two oxygen atoms from water. The Sr—O distances (Table 1) ranging from 2.475 (2) to 2.830 (2) Å, Sr polyhedra are linked into one-dimensional inorganic chain by sharing three coplanar O atoms shown as Fig. 2. The one-dimensional inorganic chains are extended along the *b* axis to form *ab* plane by O–C–O linking. The parallel layers are connected by terephthalic groups to form the three-dimensional framework, as shown in Fig. 3.

### Experimental

The suspension of the admixture Sr(NO_3_)_2_ (1 mmol) and NaOH (0.02 g) in the water (5 ml) was slowly added into the solution of terephthalic acid (2 mmol) in ethanol (10 ml) in stirred. The resulting mixture was further stirred for 4 h at 120 °C. The filtrate pH was adjusted to 3 by hydrochloric acid. The final reaction mixture was heated in a sealed Teflon-lined steel autoclave at 180 °C for 7 days. After crystallization, the autoclave was cooled down to room temperature and the yellow block single crystals were filtered, washed by distilled water and dried in air.

### Refinement

Aromatic H atoms were refined as riding atoms,with C—H=0.93Å and H atoms were calculated as *U*_iso_(H) = 1.2Ueq(carrier C). The H atoms of water were fixed in the refinements, with *U*_iso_(H)=1.5Ueq(carrier O)

### Crystal data

**Table:**

[Sr(C_8_H_4_O_4_)(H_2_O)]	*F*(000) = 1056
*M**_r_* = 269.75	*D*_x_ = 2.110 Mg m^−^^3^
Orthorhombic, *P**b**c**a*	Mo *K*α radiation, λ = 0.71073 Å
Hall symbol: -P 2ac 2ab	Cell parameters from 1205 reflections
*a* = 11.8724 (3) Å	θ = 2.7–25.2°
*b* = 7.1308 (1) Å	µ = 6.34 mm^−^^1^
*c* = 20.0592 (4) Å	*T* = 296 K
*V* = 1698.21 (6) Å^3^	Block, yellow
*Z* = 8	0.24 × 0.21 × 0.19 mm

### Data collection

**Table:**

Bruker APEXII CCD diffractometer	1523 independent reflections
Radiation source: fine-focus sealed tube	1205 reflections with *I* > 2σ(*I*)
graphite	*R*_int_ = 0.043
phi and ω scans	θ_max_ = 25.2°, θ_min_ = 2.7°
Absorption correction: multi-scan (*SADABS*; Bruker, 2001)	*h* = −14→8
*T*_min_ = 0.238, *T*_max_ = 0.300	*k* = −8→8
6767 measured reflections	*l* = −16→23

### Refinement

**Table:**

Refinement on *F*^2^	Primary atom site location: structure-invariant direct methods
Least-squares matrix: full	Secondary atom site location: difference Fourier map
*R*[*F*^2^ > 2σ(*F*^2^)] = 0.027	Hydrogen site location: inferred from neighbouring sites
*wR*(*F*^2^) = 0.062	H atoms treated by a mixture of independent and constrained refinement
*S* = 1.04	*w* = 1/[σ^2^(*F*_o_^2^) + (0.0293*P*)^2^ + 0.3991*P*] where *P* = (*F*_o_^2^ + 2*F*_c_^2^)/3
1523 reflections	(Δ/σ)_max_ = 0.001
133 parameters	Δρ_max_ = 0.36 e Å^−^^3^
3 restraints	Δρ_min_ = −0.50 e Å^−^^3^

### Special details

**Table:**

Experimental. Aromatic H atoms were refined as riding atoms,with C—H=0.93Å and H atoms were calculated as *U*_iso_(H) = 1.2Ueq(carrier C). The H atoms of water were fixed in the refinements, with *U*_iso_(H)=1.5Ueq(carrier O)
Geometry. All e.s.d.'s (except the e.s.d. in the dihedral angle between two l.s. planes) are estimated using the full covariance matrix. The cell e.s.d.'s are taken into account individually in the estimation of e.s.d.'s in distances, angles and torsion angles; correlations between e.s.d.'s in cell parameters are only used when they are defined by crystal symmetry. An approximate (isotropic) treatment of cell e.s.d.'s is used for estimating e.s.d.'s involving l.s. planes.
Refinement. Refinement of *F*^2^ against ALL reflections. The weighted *R*-factor *wR* and goodness of fit *S* are based on *F*^2^, conventional *R*-factors *R* are based on *F*, with *F* set to zero for negative *F*^2^. The threshold expression of *F*^2^ > σ(*F*^2^) is used only for calculating *R*-factors(gt) *etc*. and is not relevant to the choice of reflections for refinement. *R*-factors based on *F*^2^ are statistically about twice as large as those based on *F*, and *R*- factors based on ALL data will be even larger.

### Fractional atomic coordinates and isotropic or equivalent isotropic displacement parameters (Å^2^)

**Table:**

	*x*	*y*	*z*	*U*_iso_*/*U*_eq_	
Sr1	0.93510 (3)	0.61190 (4)	0.736154 (15)	0.01731 (12)	
O1	0.9182 (2)	0.2855 (3)	0.68486 (11)	0.0220 (6)	
O5	1.1323 (2)	0.4455 (3)	0.72230 (11)	0.0244 (6)	
C3	0.9508 (3)	0.2525 (5)	0.54589 (17)	0.0211 (8)	
H3A	1.0068	0.3131	0.5701	0.025*	
C7	0.7700 (3)	0.1036 (4)	0.54088 (16)	0.0214 (8)	
H7A	0.7041	0.0651	0.5618	0.026*	
C2	0.8539 (3)	0.1900 (4)	0.57787 (15)	0.0157 (7)	
C1	0.8405 (3)	0.2083 (4)	0.65225 (16)	0.0174 (8)	
C4	0.9639 (3)	0.2244 (4)	0.47790 (17)	0.0240 (9)	
H4A	1.0285	0.2671	0.4566	0.029*	
C6	0.7834 (3)	0.0740 (4)	0.47334 (15)	0.0213 (8)	
H6A	0.7269	0.0147	0.4491	0.026*	
C5	0.8814 (3)	0.1331 (4)	0.44164 (15)	0.0177 (8)	
C8	0.8981 (3)	0.0856 (4)	0.36917 (17)	0.0209 (8)	
O4	0.9836 (2)	0.1481 (3)	0.33885 (11)	0.0287 (6)	
O3	0.8280 (2)	−0.0220 (4)	0.34186 (11)	0.0306 (6)	
O2	0.7543 (2)	0.1373 (3)	0.67879 (11)	0.0244 (6)	
H1	1.169 (2)	0.446 (5)	0.6868 (9)	0.037*	
H2	1.165 (3)	0.505 (5)	0.7530 (11)	0.037*	

### Atomic displacement parameters (Å^2^)

**Table:**

	*U*^11^	*U*^22^	*U*^33^	*U*^12^	*U*^13^	*U*^23^
Sr1	0.01707 (19)	0.01812 (17)	0.0167 (2)	−0.00088 (15)	−0.00052 (14)	0.00049 (13)
O1	0.0225 (16)	0.0238 (13)	0.0197 (14)	−0.0034 (11)	−0.0046 (11)	−0.0034 (10)
O5	0.0202 (15)	0.0312 (13)	0.0217 (15)	−0.0025 (12)	0.0011 (11)	−0.0052 (11)
C3	0.018 (2)	0.0254 (17)	0.020 (2)	−0.0038 (16)	−0.0037 (16)	−0.0019 (14)
C7	0.018 (2)	0.0235 (17)	0.023 (2)	−0.0042 (17)	0.0020 (15)	−0.0003 (14)
C2	0.019 (2)	0.0128 (15)	0.0153 (18)	0.0005 (15)	−0.0006 (15)	−0.0004 (13)
C1	0.018 (2)	0.0125 (16)	0.021 (2)	0.0061 (15)	−0.0018 (16)	0.0023 (13)
C4	0.020 (2)	0.028 (2)	0.024 (2)	−0.0045 (16)	0.0041 (16)	0.0023 (16)
C6	0.022 (2)	0.0232 (17)	0.0189 (19)	−0.0050 (16)	−0.0033 (16)	−0.0031 (14)
C5	0.021 (2)	0.0184 (17)	0.0135 (18)	0.0028 (16)	−0.0005 (15)	0.0006 (13)
C8	0.025 (2)	0.0207 (18)	0.0173 (19)	0.0105 (17)	−0.0007 (16)	0.0036 (15)
O4	0.0312 (16)	0.0344 (15)	0.0207 (14)	−0.0004 (13)	0.0055 (12)	0.0060 (10)
O3	0.0283 (17)	0.0438 (16)	0.0197 (14)	−0.0007 (13)	−0.0025 (11)	−0.0107 (11)
O2	0.0207 (15)	0.0352 (14)	0.0172 (13)	−0.0034 (11)	0.0028 (11)	0.0004 (10)

### Geometric parameters (Å, °)

**Table:**

Sr1—O4^i^	2.475 (2)	C3—H3A	0.9300
Sr1—O2^ii^	2.533 (2)	C7—C6	1.380 (4)
Sr1—O1	2.553 (2)	C7—C2	1.386 (5)
Sr1—O3^iii^	2.554 (2)	C7—H7A	0.9300
Sr1—O5	2.639 (3)	C2—C1	1.506 (4)
Sr1—O5^iv^	2.645 (2)	C1—O2	1.259 (4)
Sr1—O1^iv^	2.660 (2)	C4—C5	1.383 (5)
Sr1—O4^iii^	2.830 (2)	C4—H4A	0.9300
Sr1—C8^iii^	3.049 (3)	C6—C5	1.391 (5)
Sr1—Sr1^iv^	3.9237 (3)	C6—H6A	0.9300
Sr1—Sr1^v^	3.9237 (3)	C5—C8	1.506 (4)
Sr1—H2	2.86 (3)	C8—O3	1.257 (4)
O1—C1	1.258 (4)	C8—O4	1.265 (4)
O1—Sr1^v^	2.660 (2)	C8—Sr1^vi^	3.049 (3)
O5—Sr1^v^	2.645 (2)	O4—Sr1^i^	2.475 (2)
O5—H1	0.84 (3)	O4—Sr1^vi^	2.830 (2)
O5—H2	0.84 (3)	O3—Sr1^vi^	2.554 (2)
C3—C4	1.387 (4)	O2—Sr1^vii^	2.533 (2)
C3—C2	1.391 (5)		
			
O4^i^—Sr1—O2^ii^	91.19 (8)	Sr1^iv^—Sr1—Sr1^v^	130.650 (17)
O4^i^—Sr1—O1	114.60 (7)	O4^i^—Sr1—H2	83.3 (7)
O2^ii^—Sr1—O1	79.18 (7)	O2^ii^—Sr1—H2	157.2 (3)
O4^i^—Sr1—O3^iii^	150.75 (8)	O1—Sr1—H2	83.1 (5)
O2^ii^—Sr1—O3^iii^	87.30 (8)	O3^iii^—Sr1—H2	108.1 (6)
O1—Sr1—O3^iii^	93.81 (8)	O5—Sr1—H2	17.1 (3)
O4^i^—Sr1—O5	84.31 (8)	O5^iv^—Sr1—H2	119.5 (5)
O2^ii^—Sr1—O5	140.53 (7)	O1^iv^—Sr1—H2	55.1 (4)
O1—Sr1—O5	67.51 (7)	O4^iii^—Sr1—H2	62.9 (7)
O3^iii^—Sr1—O5	114.60 (8)	C8^iii^—Sr1—H2	84.9 (6)
O4^i^—Sr1—O5^iv^	71.78 (7)	Sr1^iv^—Sr1—H2	81.4 (6)
O2^ii^—Sr1—O5^iv^	79.06 (7)	Sr1^v^—Sr1—H2	50.6 (6)
O1—Sr1—O5^iv^	157.43 (8)	C1—O1—Sr1	131.8 (2)
O3^iii^—Sr1—O5^iv^	79.26 (8)	C1—O1—Sr1^v^	125.89 (19)
O5—Sr1—O5^iv^	134.90 (7)	Sr1—O1—Sr1^v^	97.63 (7)
O4^i^—Sr1—O1^iv^	77.57 (8)	Sr1—O5—Sr1^v^	95.90 (8)
O2^ii^—Sr1—O1^iv^	144.96 (7)	Sr1—O5—H1	124 (3)
O1—Sr1—O1^iv^	135.71 (6)	Sr1^v^—O5—H1	116 (2)
O3^iii^—Sr1—O1^iv^	87.05 (8)	Sr1—O5—H2	96 (2)
O5—Sr1—O1^iv^	72.00 (7)	Sr1^v^—O5—H2	111 (2)
O5^iv^—Sr1—O1^iv^	65.91 (7)	H1—O5—H2	111.8 (18)
O4^i^—Sr1—O4^iii^	144.69 (3)	C4—C3—C2	120.0 (3)
O2^ii^—Sr1—O4^iii^	123.93 (8)	C4—C3—H3A	120.0
O1—Sr1—O4^iii^	73.27 (7)	C2—C3—H3A	120.0
O3^iii^—Sr1—O4^iii^	48.14 (8)	C6—C7—C2	120.7 (3)
O5—Sr1—O4^iii^	66.54 (7)	C6—C7—H7A	119.6
O5^iv^—Sr1—O4^iii^	114.92 (7)	C2—C7—H7A	119.6
O1^iv^—Sr1—O4^iii^	74.82 (7)	C7—C2—C3	119.3 (3)
O4^i^—Sr1—C8^iii^	155.18 (9)	C7—C2—C1	119.5 (3)
O2^ii^—Sr1—C8^iii^	107.62 (9)	C3—C2—C1	121.1 (3)
O1—Sr1—C8^iii^	85.42 (8)	O1—C1—O2	123.5 (3)
O3^iii^—Sr1—C8^iii^	23.91 (9)	O1—C1—C2	118.4 (3)
O5—Sr1—C8^iii^	90.71 (9)	O2—C1—C2	118.0 (3)
O5^iv^—Sr1—C8^iii^	95.51 (8)	C5—C4—C3	120.4 (3)
O1^iv^—Sr1—C8^iii^	77.77 (8)	C5—C4—H4A	119.8
O4^iii^—Sr1—C8^iii^	24.48 (9)	C3—C4—H4A	119.8
O4^i^—Sr1—Sr1^iv^	45.92 (5)	C7—C6—C5	119.9 (3)
O2^ii^—Sr1—Sr1^iv^	110.37 (5)	C7—C6—H6A	120.1
O1—Sr1—Sr1^iv^	156.42 (6)	C5—C6—H6A	120.1
O3^iii^—Sr1—Sr1^iv^	107.83 (6)	C4—C5—C6	119.7 (3)
O5—Sr1—Sr1^iv^	94.30 (5)	C4—C5—C8	121.3 (3)
O5^iv^—Sr1—Sr1^iv^	42.00 (6)	C6—C5—C8	118.9 (3)
O1^iv^—Sr1—Sr1^iv^	40.16 (5)	O3—C8—O4	122.5 (3)
O4^iii^—Sr1—Sr1^iv^	114.35 (5)	O3—C8—C5	118.1 (3)
C8^iii^—Sr1—Sr1^iv^	110.64 (6)	O4—C8—C5	119.4 (3)
O4^i^—Sr1—Sr1^v^	124.15 (6)	O3—C8—Sr1^vi^	55.42 (17)
O2^ii^—Sr1—Sr1^v^	118.56 (5)	O4—C8—Sr1^vi^	68.04 (18)
O1—Sr1—Sr1^v^	42.22 (5)	C5—C8—Sr1^vi^	165.4 (2)
O3^iii^—Sr1—Sr1^v^	81.37 (6)	C8—O4—Sr1^i^	148.1 (2)
O5—Sr1—Sr1^v^	42.11 (5)	C8—O4—Sr1^vi^	87.5 (2)
O5^iv^—Sr1—Sr1^v^	153.08 (5)	Sr1^i^—O4—Sr1^vi^	95.16 (7)
O1^iv^—Sr1—Sr1^v^	94.67 (5)	C8—O3—Sr1^vi^	100.7 (2)
O4^iii^—Sr1—Sr1^v^	38.92 (5)	C1—O2—Sr1^vii^	160.4 (2)
C8^iii^—Sr1—Sr1^v^	60.86 (7)		
			
O4^i^—Sr1—O1—C1	−89.6 (3)	C4—C3—C2—C1	176.3 (3)
O2^ii^—Sr1—O1—C1	−3.3 (3)	Sr1—O1—C1—O2	−80.0 (4)
O3^iii^—Sr1—O1—C1	83.2 (3)	Sr1^v^—O1—C1—O2	70.1 (4)
O5—Sr1—O1—C1	−161.7 (3)	Sr1—O1—C1—C2	103.5 (3)
O5^iv^—Sr1—O1—C1	12.3 (4)	Sr1^v^—O1—C1—C2	−106.4 (3)
O1^iv^—Sr1—O1—C1	172.9 (3)	C7—C2—C1—O1	179.3 (3)
O4^iii^—Sr1—O1—C1	127.3 (3)	C3—C2—C1—O1	1.6 (5)
C8^iii^—Sr1—O1—C1	105.6 (3)	C7—C2—C1—O2	2.6 (4)
Sr1^iv^—Sr1—O1—C1	−119.9 (3)	C3—C2—C1—O2	−175.2 (3)
Sr1^v^—Sr1—O1—C1	155.9 (3)	C2—C3—C4—C5	−0.5 (5)
O4^i^—Sr1—O1—Sr1^v^	114.47 (9)	C2—C7—C6—C5	−0.7 (5)
O2^ii^—Sr1—O1—Sr1^v^	−159.22 (9)	C3—C4—C5—C6	1.8 (5)
O3^iii^—Sr1—O1—Sr1^v^	−72.71 (8)	C3—C4—C5—C8	−174.5 (3)
O5—Sr1—O1—Sr1^v^	42.38 (7)	C7—C6—C5—C4	−1.2 (5)
O5^iv^—Sr1—O1—Sr1^v^	−143.65 (15)	C7—C6—C5—C8	175.1 (3)
O1^iv^—Sr1—O1—Sr1^v^	16.97 (6)	C4—C5—C8—O3	169.2 (3)
O4^iii^—Sr1—O1—Sr1^v^	−28.62 (7)	C6—C5—C8—O3	−7.0 (4)
C8^iii^—Sr1—O1—Sr1^v^	−50.29 (9)	C4—C5—C8—O4	−7.9 (5)
Sr1^iv^—Sr1—O1—Sr1^v^	84.17 (13)	C6—C5—C8—O4	175.8 (3)
O4^i^—Sr1—O5—Sr1^v^	−162.10 (8)	C4—C5—C8—Sr1^vi^	109.6 (10)
O2^ii^—Sr1—O5—Sr1^v^	−77.17 (13)	C6—C5—C8—Sr1^vi^	−66.7 (11)
O1—Sr1—O5—Sr1^v^	−42.49 (7)	O3—C8—O4—Sr1^i^	84.8 (5)
O3^iii^—Sr1—O5—Sr1^v^	41.17 (9)	C5—C8—O4—Sr1^i^	−98.2 (5)
O5^iv^—Sr1—O5—Sr1^v^	140.77 (9)	Sr1^vi^—C8—O4—Sr1^i^	95.7 (4)
O1^iv^—Sr1—O5—Sr1^v^	119.15 (8)	O3—C8—O4—Sr1^vi^	−11.0 (3)
O4^iii^—Sr1—O5—Sr1^v^	38.30 (6)	C5—C8—O4—Sr1^vi^	166.1 (3)
C8^iii^—Sr1—O5—Sr1^v^	42.26 (8)	O4—C8—O3—Sr1^vi^	12.4 (4)
Sr1^iv^—Sr1—O5—Sr1^v^	153.01 (5)	C5—C8—O3—Sr1^vi^	−164.7 (2)
C6—C7—C2—C3	2.0 (5)	O1—C1—O2—Sr1^vii^	74.7 (7)
C6—C7—C2—C1	−175.8 (3)	C2—C1—O2—Sr1^vii^	−108.7 (6)
C4—C3—C2—C7	−1.4 (5)		

Symmetry codes: (i) −*x*+2, −*y*+1, −*z*+1; (ii) −*x*+3/2, *y*+1/2, *z*; (iii) *x*, −*y*+1/2, *z*+1/2; (iv) −*x*+2, *y*+1/2, −*z*+3/2; (v) −*x*+2, *y*−1/2, −*z*+3/2; (vi) *x*, −*y*+1/2, *z*−1/2; (vii) −*x*+3/2, *y*−1/2, *z*.

### Hydrogen-bond geometry (Å, °)

**Table:**

*D*—H···*A*	*D*—H	H···*A*	*D*···*A*	*D*—H···*A*
O5—H1···O3^viii^	0.84 (3)	2.03 (4)	2.711 (3)	137 (3)
O5—H2···O2^iv^	0.84 (3)	1.92 (3)	2.761 (3)	178 (3)

Symmetry codes: (viii) *x*+1/2, −*y*+1/2, −*z*+1; (iv) −*x*+2, *y*+1/2, −*z*+3/2.
